# Immunohistochemical and morphological features of a small bowel leiomyoma in a black crested macaque (*Macaca nigra*)

**DOI:** 10.1186/1746-6148-8-97

**Published:** 2012-06-29

**Authors:** Mónica Aristizabal-Arbelaez, Julian Mejía-Restrepo, Mauricio Montoya-Flórez, Fabrizio Grandi, Francisco Pedraza-Ordóñez

**Affiliations:** 1 Veterinary Pathology Research Group, Faculty of Agricultural Sciences, Universidad de Caldas, Manizales, Colombia; 2 Laboratory of Investigative and Comparative Pathology, Department of Veterinary Clinics, Universidade Estadual Paulista, UNESP, Botucatu, Brazil; 3 Animal Health Department, Universidad de Caldas, Manizales, Colombia; 4 Department of Pathology, Botucatu Medical School, Universidade Estadual Paulista, UNESP, Botucatu, Brazil; 5 Department of Diagnostic Pathology, Public Veterinary Hospital, Veterinary Service of National Association of Small Animal Clinicians (Anclivepa), São Paulo, SP, Brazil

**Keywords:** Neoplasm, Small bowel, *Macaca*, Monkey, Leiomyoma, Intestine

## Abstract

**Background:**

Spontaneous gastrointestinal neoplasms in non-human primates are commonly seen in aged individuals. Due to genetic similarities between human and non-human primates, scientists have shown increasing interest in terms of comparative oncology studies.

**Case presentation:**

The present study is related to a case of an intestinal leiomyoma in a black crested macaque (*Macaca nigra*), kept on captivity by Matecaña Zoo, Pereira City, Colombia. The animal had abdominal distension, anorexia, vomiting, diarrhea and behavioral changes. Clinical examination showed an increased volume in the upper right abdominal quadrant caused by a neoplastic mass. The patient died during the surgical procedure. Necropsy revealed several small nodules in the peritoneum with adhesion to different portions of the small and large intestines, liver, stomach and diaphragm. Tissue samples were collected, routinely processed and stained by H&E. Microscopic examination revealed a mesenchymal tumor limited to *tunica muscularis,* resembling normal smooth muscle cells. Neoplastic cells were positive for alpha-smooth muscle actin and vimentin, and negative for cytokeratin AE1/AE3 by immunohistochemistry. Those morphological and immunohistochemical findings allowed to diagnose the intestinal leiomyoma referred above.

**Conclusion:**

Neoplastic diseases in primates have multifaceted causes. Their manifestations are understudied, leading to a greater difficulty in detection and measurement of the real impact provides by this disease.

## Background

According to some surveys, Spontaneous gastrointestinal neoplasm in non-human primates is the most common tumor described in literature, accounting for up to 48.8% of the cases [1]. The disease is commonly seen in aged animals [2,3] which has caused an increasing scientific interest, in terms of comparative oncology studies, due to genetic similarities between human and non-human primates [1].

Intestinal adenocarcinoma is the most prevalent tumor reported in primates belonging to *Macaca* genus [1], constituting a substantial cause of morbidity and mortality in the elderly [4]. However, other kind of tumor such as schwannoma [5], gastrointestinal stromal [6,7], leiomyosarcoma [8] and a poorly differentiated sarcoma [9], were also reported.

Leiomyoma, after adenoma, is the most common tumor of the small bowel in human [10,11], which is usually detected incidentally on necropsy evaluation or non-related surgical procedures [12]. Although described in human [12] and other non-human primates such as dwarf galagos (*Galagoides demidovii*) and cotton-tap tamarins (*Saguinus oedipus*) [13], a specific case related to *Macaca sp*. was not found, up to date.

The present study describes clinical signs, biochemical and hematological parameters, necropsy and microscopic findings of a case related to a spontaneous intestinal leiomyoma in a crested black macaque.

## Case presentation

**Figure 1:**
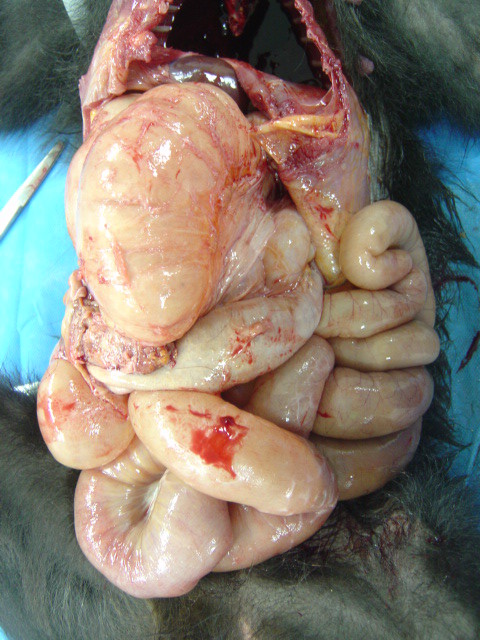
**Macroscopic aspect of Leiomyoma in small bowel of the*Macaca nigra*.** Accumulation of gas in the intestinal loops and presence of a mass (circle) in the large bowel.

A 6-year-old female crested black macaque, born on captivity in Matecaña Zoo, Pereira City, Colombia was presented at Veterinary Hospital for clinical evaluation. Reportedly, it was suffering from isolation, apathy, weakness, anorexia, and recurrent episodes of diarrhea, with loose stools, and vomiting. At first, a regular corporal condition of the animal was determined (2.5; interval range: 1 to 5), with a weight of 6 kg. However, some clinical disorders as pale mucous membranes, weak pulse and mild hypothermia were presented. Auscultation revealed rattles at the lower respiratory tract and increased peristalsis. Palpation allowed detecting a marked abdominal distention mainly in the upper right quadrant caused by a firm and irregular intra-abdominal mass. Ultrasound evaluation confirmed the presence of an irregular, multilobulated mass measuring 3.0 x 5.0 x 3.0 cm, attached to internal organs. Other organs were not properly visualized because of an excessive gas accumulation in the small and large bowel (Figure 1).

Hematological profile revealed leukocytes, showing prominent neutrophilia and eosinophilia and jaundiced plasma (Table 1). Biochemical profile revealed increase in BUN, serum creatinin, alanine aminotransferase (ALT), aspartate aminotransferase (AST) and alkaline phosphatase levels (Table 2). Other ancillary tests were not performed.

**Table 1:** Results of hematological profile

**Parameter**	**Result**		Reference value*
Hematocrit	34%		26-44.4
Hemoglobin	10.56 g/dl		8,4-12.8
Red cell blood count	4.3 x10^12^/l		3,8-6.4
MCV**	78.4 fl		59.3-75.7
MCHC**	31.1 g/dl		26.4-33.6
Total protein	8.2 g/dl		5.3-8.5
White blood cell count	30350 μl		3090-17070
Neutrophils	24584 μl	81%	408-12336
Neutrophils bands	---------		948-2712 μl
Lymphocytes	3339 μl	11%	1307-7537
Monocytes	1214 μl	4%	1001-2195
Eosinophiles	1214 μl	4%	239-581
Platelet count	260100 mm^3^		249000-631000

Reference values were taken from the International Species Information System: Physiological Norms, Apple Valley, Minn., 2002, ISIS.

** MCV, mean corpuscular volume, MCHC, mean corpuscular hemoglobin concentration.

**Table 2:** Results of serum biochemical profile

**Parameter**	**Result**	Reference range*
BUN**	32.8 mg/dl	0.6-5.4
Creatinine	2.3 mg/dl	0.4-1.2
Alkaline phosphatase	1853U/l	294-1402
ALT**	453 U/l	5-81
AST**	568.6 U/l	5-109
GGT**	19.5 U/l	6-290
Albumine	2.3 g/dl	2.3-5.5

Reference values were taken from the International Species Information System: Physiological Norms, Apple Valley, Minn., 2002, ISIS.

** BUN, blood urea nitrogen, ALT, alanine aminotransferase, AST, aspartate aminotransferase, GGT, gamma glutamyl transferase.

Tissue samples from the mass were collected by an exploratory laparotomy in order to conduct further histopathological evaluation. However, during this surgical procedure, the animal died because of cardiac arrest, being immediately evaluated by a necropsy.

### Necropsy findings

As a result of macroscopic evaluation, gross changes as a shaggy coat and dry skin were found. Also, a marked abdominal distension and an easily palpable intra-abdominal mass were located in the upper right quadrant. Subcutaneous tissue showed a marked yellow discoloration (jaundice).

Peritoneal cavity was full-filled with an evident quantity of a hemorrhagic fluid. Peritoneal surface was thickened by scattered multifocal coalescent grayish-white, with firm and well defined nodules of variable sizes. There were multiple adhesions between the serosal surfaces of liver, stomach, peritoneum and diaphragm. A firm, multilobulated, non-encapsulated, poor defined grayish-white mass measuring 3.0x 5.0 cm x 3.0 cm, originated from the final portion of a stenotic ileum, filled the entire upper right quadrant. The exact origin within the ileum was not possible to determine. Stomach, small intestine and large intestine were distended by abundant gas. Pancreas showed small white nodules on surface. Spleen showed several small white dots on cut surface. The mesenteric lymph nodes were increased in size and thickness. Also, their consistency was firm. Samples from all affected organs were fixed in 10% neutral-buffered formalin solution, routinely processed and stained with hematoxylin and eosin (H&E) at the Laboratory of Pathology, Universidad de Caldas, Colombia.

### Microscopic findings

Histopathological examination of small bowel mass tissue sections revealed an expanded, well delimited, non-encapsulated, highly cellular mesenchymal neoplasm, resembling normal smooth muscle cells, which were arranged in multidirectional fascicles and restricted to *tunica muscularis*. These cells were fusiform, having both an indistinct cell boundaries and an intensely and homogeneous eosinophilic cytoplasm. Sometimes, they displayed perinuclear clearing. The nuclei were elongated, cigar-shaped with a finely stippled chromatin pattern and a single central nucleolus. Mitotic figures were rarely seen. As a result of these findings, a small bowel leiomyoma was diagnosed. Liver revealed a thickened capsule. Its portal areas were infiltrated by a mild lymphoplasmacytic. Likewise, several intracytoplasmic clear vacuoles in centrilobular hepatocytes were found. Lungs had diffuse congestion, anthracnose and severe multifocal emphysema. Heart had a large amount of fibrillar and intense acidophilic material (fibrin clots) attached to epicardial surface. Kidneys showed a nephropathy, characterized by a decrease of Bowman’s space of some renal corpuscles. Also, dilatation and congestion of the peritubular vessels and a discrete intratubular protein deposit were present.

### Immunohistochemistry

Immunohistochemistry was performed at Laboratory of Investigative and Comparative Pathology, Universidade Estadual Paulista, Botucatu, São Paulo, Brazil. Briefly, 4 mm histological sections were obtained in positive charged slides (Amitel®), which were deparaffinized in xylene and rehydrated in decreased ethanol concentrations. Tissue sections were exposed to heat in a Pascal chamber (Dako® Cytomation) with citrate buffer solution (pH 7.0) at 125°C for 30 sec in order to induce them to epitope retrieval (HIER). The slides were rinsed 3x by mean of Tris pH7.0. Then, they were incubated (overnight) by using primary antibodies such as vimentin, α-SMA, and cytokeratin AE1/AE3 (Table 3). Negative controls were prepared by substituting specific primary antibodies with antibody diluent (Novocastra®). The resulting reaction was visualized with a polymer based technology HiDef® Detection HRP System (Cell Marque®). The slides were revealed with chromogen 3,3´-diaminobenzidine (DAB). Then, they were counter-stained by using Harris hematoxylin.

**Table 3:** Specific dilutions and sources of primary antibodies

**Antibody (clone)**	**Dilution**	**Source**	**Clonality/host**
Cytokeratin (AE1/AE3)	1:500	Dako Cytomation®	Monoclonal mouse
Vimentin (A9)	1:2000	Dako Cytomation ®	Monoclonal mouse
α-SMA (CGA-7)	1:2000	Santa Cruz Biotechnology®	Monoclonal mouse

**Figure 2:**
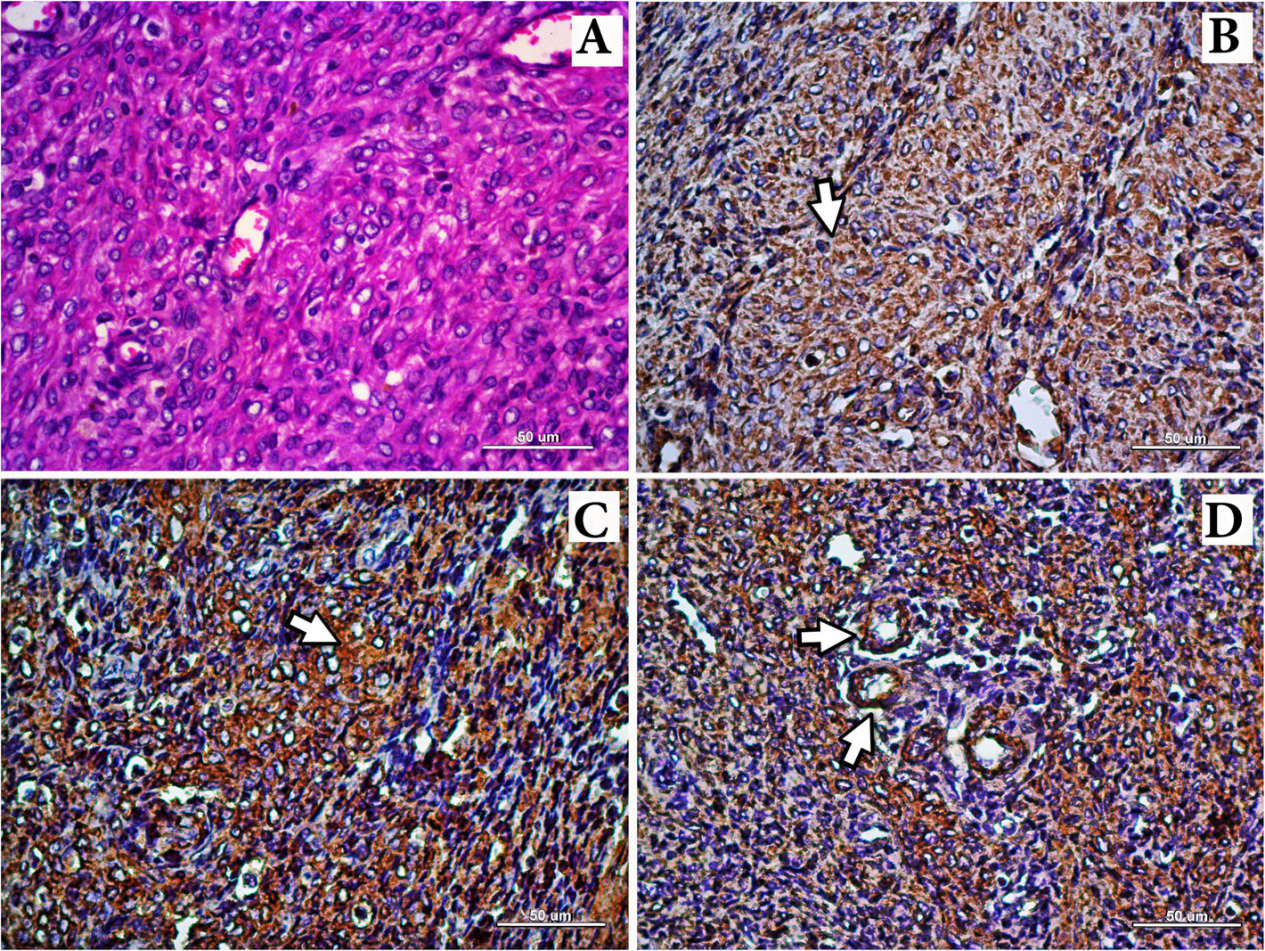
**Microscopic aspect of Leiomyoma in small bowel of the*Macaca nigra*.A**. Small bowel mass, resembling normal smooth muscle cells arranged in multidirectional fascicles, H&E, 40x, Bar: 50 μm. **B**. Moderate and diffuse cytoplasmic immunolabeling for vimentin in a fascicle of neoplastic smooth muscle cells (white arrow). DAB chromate (3,3´-diaminobenzidine), 40x, Bar 50 μm. **C**. Intense and diffuse cytoplasm tic immunolabelling for α-SMA in neoplastic cells (white arrow). DAB chromogen (3,3´-diaminobenzidine), 40x, Bar: 50 μm. **D**. Intense and diffuse cytoplasmatic immunolabelling for α-SMA in neoplastic cells showing an internal control (small arterioles, white arrows). DAB chromogen (3,3´-diaminobenzidine), 40x, Bar: 50 μm.

All neoplastic muscle cells showed an intense, homogeneous and diffuse cytoplasmic vimentin and α-smooth muscle actin immunolabeling (Figure 2); neoplastic cells were negative for cytokeratin AE1/AE3.

## Discussion

The crested black macaque (*Macaca nigra*) is an Old World primate that lives in the rainforest of Sulawesi Island, Indonesia. Nowadays, it is considered one of the most endangered species [14].

There are several reports regarding intestinal neoplasms in primates. A study on prevalence of spontaneous neoplasm in two colonies of Captive Rhesus Macaques found that the gastrointestinal system was the most commonly affected. It also established adenocarcinoma as the most prevalent identified tumor [1]. Other study described carcinomas in 2 rhesus macaques (*M. mulatta*) and 4 common marmosets [15]. Regarding to leiomyoma in non-human primates, some research have described their findings at gastrointestinal level in dwarf galagos (*Galagoides demidovi*i) and cotton-tap tamarins (*Saguinus oedipu*s) [13]. A research conducted in two populations of Captive Rhesus Macaques (*Macaca mulatta*), one leyomioma in stomach was differenced from 127 gastrointestinal diagnosed tumors [1]. Regarding females of chimpanzees, the most common tumor was the leiomyoma of the uterus, cervix and vagina [16]. Referred to Rhesus Macaques (*Macaca mulatta*), from 61 tumors diagnosed in uterus, 30 of them corresponded to leiomyoma [1]. A review compiling 247 neoplasms founded in 217 prosimians, three leiomyoma were described in uterus (two in Coquerel’s giant mouse lemur and one in Mongoose lemur [17]). Finally, a case of splenic angioleiomyoma was discovered in an Owl monkey (*Aotus nancymae*) as a rare benign neoplasm [18]. The epidemiologic studies on small bowel tumors are limited, in part, due to their low incidence [10], or by the low susceptibility of this anatomical region to neoplasm [15]. According to Lu *et al*., 2012 [19], in human medicine, tumor in small bowel is rare, representing only 5% of gastrointestinal tumor cases. Likewise, small bowel rarely develops malignant tumors [20]. Fewer than 2% of all GI malignancies are originated in the small bowel [21]. In relation to benign tumor in small bowel, leiomyoma is the most common type [10], representing 20 to 30% of tract tumors [22]. Some analysis showed that leiomyomas account for almost one fourth of all benign tumors in small bowel [12]. Therefore, the low reporting of benign tumor in small intestine of non-human primates is due to similar behavior of neoplasm in human.

The most common clinical symptoms associated with small bowel tumor are related to obstruction and bleeding. Obstruction usually manifests as recurrent crampy abdominal pain. The pain is usually periumbilical or epigastric, occurring after meals. Some people may experience associated bloating, nausea, or vomiting [10]. However, in many cases, the diagnosis is difficult due to the absence of symptoms [22]. In a retrospective analysis conducted on 10 common marmosets (*Callithrix jacchus*) suffering from small intestinal adenocarcinoma, nine of them had common symptoms related to weight loss and an intractable diarrhea, with variable presence of blood [15]. Human patients from other reports showed symptoms of anorexia, and recurrent episodes of diarrhea with loose stools and vomiting.

Related to present study, patient´s symptoms such as anorexia, and recurrent episodes of diarrhea, with loose stools, and vomiting, match tract tumor cases reported in previous studies and revisited bibliography. Moreover, abdominal bloating symptom coincides with an accumulation of gas produced by a tumor intestinal obstruction, among other causes, as previously found in human [23-25]. In other words, a relationship between those symptoms and bowel tumor can be established.

Lower gastrointestinal tract tumor is uncommon in domestic animal, especially in horse and production animals. However, this tumor has been found frequently in surgical biopsy applied to dogs and cats. Malignant neoplasm is more common than benign tumor. With the exception of lymphosarcoma, carcinomas are the most [26]. In human, a variety of infectious, genetic and environmental factors may contribute to gastrointestinal tumorigenesis. It is well known that *Helicobacter sp. *bacteria is involved in the development of gastric and colonic carcinoma in human and immunodeficient mouse. Isolation of this species of bacteria in cotton-toptamarins (*Saguinus oedipus*), suffering from chronic colitis, showed no connection with a potential tumor formation [15]. On the other hand, different members of the family Herpesviridae can affect primates, having different biological characteristics, and producing latent infections in secretion glands, lymphoid organs, kidneys and other tissues. In addition to, they can produce irreversible cytolitic effects and severe systemic inflammatory reactions [27]. Epstein-Barr virus, a human herpes virus has been commonly associated with lymphoproliferative diseases including lymphosarcoma and gastric adenocarcinomas. The Epstein-Barr related virus such as Callitrichine Herpesvirus 3 (CHV-3) has been isolated from common marmosets with lymphosarcoma, but an association with other malignancies in these marmosets has not been determined [15]. Non-human primate is very popular in zoological collections and also an important focus for the social sciences and biomedical research. Direct and indirect contact associated with this animal have repeatedly demonstrated the movement of major infectious diseases related to an interspecies manner. The prevention to infectious disease exposure is important and fundamental for the conservation of primates [28].

In human, there is a genetic predisposition to gastrointestinal cancer due to a defect in repair genes, or DNA mismatch repair genes (MMR), causing hereditary nonpolyposis colorectal cancer (HNPCC). Affected individuals are predisposed to develop neoplasms, and more frequently develop adenocarcinomas of the colon [29]. As a support for environmental carcinogens, epidemiological studies have been performed, which suggest relationships between the development of cancer and exposure to picolinic acid herbicides and phenoxides and high population densities. On the other hand, the ingestion of diets high in fat constitutes a major risk factor for colorectal cancer [29]. Based on genetic similarities between human and non-human primates, it is important to gather and study several cases of gastrointestinal neoplasms in order to establish a new spontaneous animal model for future comparative surveys.

Immunohistochemical study revealed neoplastic cells negative for cytokeratin, and positive for vimentin and α-smooth muscle actin, which his consistent with an IHC profile of a smooth muscle neoplasm. Despite this, there are other immunohistohemical markers that could be useful in this case such as S-100, CD34 and CD117 (c-KIT). The c-KIT is expressed in gastrointestinal stromal tumor (GIST) [30,31] together with CD34 as double-positive reactivity [32] and therefore must be taken into account in the differential diagnosis. S-100 antibody is a marker of neurogenic differentiation, lacking of leiomyomas [30]. Unfortunately, we are unable to test immunoreactivity to c-KIT, CD34 and S-100 due to loss of the remaining paraffin embedded tissue during transport between Colombia and Brazil. Thus, in future assays, the use of these markers would be very important in order to classify more accurately primary intestinal tumors.

The hematological examination revealed leukocytes, exhibiting prominent neutrophilia and eosinophilia. Neutrophilia is a common indicator for an inflammatory response to an infectious or non-infectious origin. Likewise, eosinophilia is characteristic of allergic inflammation or parasites [33]. Both, neutrophilia and eosinophilia, are usually considered as indicators of a parasitic infection [34]. Despite of, no parasite was identified in the necropsy and microscopic evaluation.

The serum chemistry parameters reached increased levels of ALT, AST and ALP, which collectively indicate liver injury. This was confirmed by histopathological analysis of the liver that revealed lymphoplasmacytic infiltrate in addition to several intracytoplasmic vacuolation (steatosis). By itself, creatinine is formed in the muscle during the metabolism of creatine. Then, it is subsequently diffuse into the blood, which is transported to the kidney, where is filtered by the glomerulus and, finally, is excreted in the urine [35]. Creatinine once formed in the muscle cannot longer be re-converted into creatine, its predecessor. Thus, this metabolite is used as an indicator of muscle metabolism and renal function. The increased level of this substance in the patient blood suggested alteration in muscle or kidney. Finally, once the kidney was analyzed by means of histological procedures, the alteration suffered by the organ was confirmed. In other words, the increase of creatinine was caused by a kidney failure of the animal.

## Conclusions

Development of malignant neoplasms in general is due to a genetic predisposition and exposure to risk factors such environmental carcinogen. However, chronic inflammation and viral or bacterial gene mutations also could play an important role in promoting tumor development in both, human and animal.

Neoplastic diseases in primates have multifaceted causes. Their behaviors are understudied leading to a greater difficulty in the detection and measurement of the real impact that this disease provides. The information of the basic factors that influence this disease such dietary habits, nutritional requirements, behavior, normal biochemical values and susceptibility to various agents is poorly understood. The understanding of the disease requires more detailed research efforts in order to understand pathogenesis and to create management plans [36] to improve animal and human welfare, which is, in fact, the ultimate goal of the medical field.

## Abbreviations

ALT: Alanine aminotransferase; AST: Aspartate aminotransferase; ALP: Alkaline phosphatase; BUN: Blood urea nitrogen; DAB: Chromogen 3,3´-diaminobenzidine; CHV-3: Callitrichine Herpesvirus 3; HNPCC: Causing hereditary nonpolyposis colorectal cancer; CD34: Cluster differentiation; GGT: Gamma glutamyl transferase; H&E: Hematoxylin and eosin; HIER: Heat induced epitope retrieval; IHC: Immunohistochemical; MCV: Mean corpuscular volume; MCHC: Mean corpuscular hemoglobin concentration; MMR: Mismatch repair genes; α-SMA: α smooth muscle actin; S-100: S-100 protein; c-KIT: Tyrosine protein kinase Kit.

## Competing interest

None of the authors of the present document has conflict of interest for its publication.

## Authors’ contributions

JMR: Clinical management of the case and elaboration of the preliminary report FPO, MMF, MAA: Analysis and interpretation of data, making and critical review of the manuscript. FG: Imunohistochemical analysis and critical review of the manuscript. All authors read and approved the final manuscript.
